# Increased Levels of Autoantibodies against ROS-Modified Proteins in Depressed Individuals with Decrease in Antibodies against SARS-CoV-2 Antigen (S1-RBD)

**DOI:** 10.3390/cimb44110358

**Published:** 2022-10-28

**Authors:** Subuhi Sherwani, Mohamed Raafat, Saravanan Rajendrasozhan, Mahvish Khan, Mohd Saleem, Qayyum Husain, Saif Khan, Noor Alam, Mohd Wajid Ali Khan

**Affiliations:** 1 Department of Biology, College of Sciences, University of Ha’il, Ha’il 2440, Saudi Arabia; 2 Department of Physiotherapy, College of Applied Medical Sciences, University of Ha’il, Ha’il 2440, Saudi Arabia; 3 Department of Chemistry, College of Sciences, University of Ha’il, Ha’il 2440, Saudi Arabia; 4 Department of Pathology, Sub-Division of Medical Microbiology, College of Medicine, University of Ha’il, Ha’il 2440, Saudi Arabia; 5 Department of Biochemistry, Faculty of Life Sciences, Aligarh Muslim University, Aligarh 202002, India; 6 Department of Basic Dental and Medical Sciences, University of Ha’il, Ha’il 2440, Saudi Arabia; 7 Department of Mathematics, College of Sciences, University of Ha’il, Ha’il 2440, Saudi Arabia; 8 Molecular Diagnostics and Personalized Therapeutics Unit, University of Ha’il, Ha’il 2440, Saudi Arabia

**Keywords:** COVID-19, SARS-CoV-2, depression, oxidative stress, ROS, ELISA, autoantibody, S1-RBD, S1-RBD-Abs

## Abstract

Coronavirus 2019 (COVID-19) disease management is highly dependent on the immune status of the infected individual. An increase in the incidence of depression has been observed during the ongoing COVID-19 pandemic. Autoantibodies against in vitro reactive oxygen species (ROS) modified BSA and Lys as well as antibodies against receptor binding domain subunit S1 (S1-RBD) (S1-RBD-Abs) of severe acute respiratory syndrome coronavirus 2 (SARS-CoV-2) were estimated using direct binding and competition ELISA. Serum samples were also tested for fasting blood glucose (FBG), malondialdehyde (MDA), carbonyl content (CC), interferon-gamma (IFN-γ) and tumor necrosis factor-alpha (TNF-α). Significant structural changes were observed in ROS modified BSA and Lys. Female depressed subjects who were also smokers (F-D-S) showed the highest levels of oxidative stress (MDA and CC levels). Similarly, increased levels of autoantibodies against ROS modified proteins were detected in F-D-S subjects, in males who were depressed and in smokers (M-D-S) compared to the other subjects from the rest of the groups. However, contrary to this observation, levels of S1-RBD-Abs were found to be lowest in the F-D-S and M-D-S groups. During the pandemic, large numbers of individuals have experienced depression, which may induce excessive oxidative stress, causing modifications in circulatory proteins. Thus, the formation of neo-antigens is induced, which lead to the generation of autoantibodies. The concomitant effect of increased autoantibodies with elevated levels of IFN-γ and TNF-α possibly tilt the immune balance toward autoantibody generation rather than the formation of S1-RBD-Abs. Thus, it is important to identify individuals who are at risk of depression to determine immune status and facilitate the better management of COVID-19.

## 1. Introduction

COVID-19 is a contagious viral disease caused by the single-stranded RNA SARS-CoV-2 [1]. In November 2019, the virus was reportedly detected in Wuhan, China [2]. By early 2020, COVID-19 had rapidly spread to all continents and was subsequently declared a global pandemic in March 2020 by the World Health Organization [3]. The disease has had a far-reaching impact on the social, health and economic lives of populations the world over [4]. Infected individuals have cold-like symptoms with wide-ranging complications, such as respiratory distress, multi-organ failure and even death, because of inflammatory changes brought on by a cytokine storm in patients with comorbid conditions [5]. 

Antibodies against SARS-CoV-2 infection are necessary to combat the virus, as substantial levels of antibody response may effectively counter the number of virions that can target the receptor angiotensin converting enzyme-2 (ACE-2)-expressing cells [6]. Thus, it is well understood that COVID-19-specific antibodies play a crucial role and become an important line of defense during infection [7]. Antibody titer have been associated with protection against SARS-CoV-2 infection [8,9]. COVID-19 is characterized by the overproduction of pro-inflammatory cytokines, linked to adverse patient outcomes and patient mortality. Of the many inflammatory cytokines produced in SARS-CoV-2 infection, it has been found that TNF-α and IFN-γ contribute to inflammatory cell death [5,10]. A correlation was found between neutralizing antibodies, severity of COVID-19 infection and increased levels of pro-inflammatory cytokines and chemokines [11]. Dysregulation of immune responses together with increased inflammatory conditions might affect the production and levels of these antibodies. 

Lifestyle changes enforced and adopted throughout the world, such as lockdowns, quarantine and working from home during the first wave, and then through successive outbreaks have since contributed to changes in behavior, decreases in routine physical activity, modifications to diet and a range of mental health and psychological problems like insomnia, stress, anxiety and depression [12,13]. Depression is a common mood disorder, which affects the normal day-to-day functioning of individuals. Symptoms can range from mild to severe, impacting every sphere of life [14]. Some risk factors that contribute to the incidence of depression include genetic, biological, psychological and environmental [15]. Research suggests that mentally stressed individuals are at risk of developing physical illnesses [15] and diseases such as obesity [16], diabetes mellitus [17], heart disease [18] and cancer [19].

A study on depression suggested that the worldwide occurrence of depression increased seven times from 3.44% in 2017 to 25% during the COVID-19 pandemic, indicating an adverse effect on individuals’ mental health [20]. Several studies conducted during the pandemic indicate that COVID-19 has fueled an increase in fear, anxiety, stress, and depression among different student populations too [21,22]. 

In a meta-analysis study, the levels of oxidative markers 8-hydroxy-2′-deoxyguanosine and F2-isoprostanes, which are specific for DNA and lipid damage, respectively, were found to increase in depression [23]. Mental stress in individuals may contribute to increased metabolic and oxidative stress and subsequent cellular damage, eventually leading to the pathogenesis of disease [24]. Research suggests a close relationship between the development of depression and the enhancement of oxidative and nitrosative stress [25,26]. The increased production of ROS and reactive nitrogen species (RNS) exerts irreversible cellular damage, resulting in cell death [27]. Excessively produced ROS/RNS might create an imbalance in redox homeostasis [27] and lead to structural modification in biomolecules (proteins/DNA). Furthermore, it is believed that smoking aggravates oxidative stress due to various mechanisms, including an increase in free radicals’ formation and inflammatory responses [28].

There were several studies wherein autoantibodies were detected in COVID-19 patients [29]. Zhou et al. demonstrated the presence of antinuclear antibodies (ANAs) in 50% of severely and critically ill COVID-19 patients [30]. Another study by Vlachoyiannopoulos et al. showed 34.5% of ANA positivity in severely ill COVID-19 patients [31]. Pascolini et al. also found a 33.3% positivity for ANAs in COVID-19 patients [32]. Chronic low-grade inflammatory conditions inducing autoantibody production have been implicated in physiological conditions in depression [25,26,33]. Patients of depression with chronic diseases (systemic lupus erythematosus, rheumatoid arthritis, and type 1 diabetes) exhibit higher amounts of various specific autoantibodies [25,26,33,34]. Evaluation of humoral response in normal and depressed individuals is crucial to protecting this vulnerable population, at a higher risk of responding poorly to infection with and vaccination against SARS-CoV-2. Despite the limitations of a small number of recruited participants in this study, we believe our results are important, showing that psychologically depressed individuals may be at a higher risk of poor response to SARS-CoV-2 infection. 

According to our hypothesis, during depression, there is increased production of ROS and RNS, which may lead to structural modifications in biomolecules (proteins/DNA). Damage to biomolecules may be further aggravated in smokers. These modified biomolecules may behave as autoantigens, against which autoantibodies are generated. Thus, oxidative stress is an important factor in the development of several diseases. 

The present study was designed to investigate the effects of psychological and oxidative stress on the levels of antibodies during the COVID-19 pandemic. The detection of S1-RBD-Abs, as well as autoantibodies against other free-radical-modified antigens (ROS-BSA and ROS-Lys), may lead to a better understanding of immune imbalances under psychological stress during the pandemic. This is a unique community-based study in this region.

## 2. Materials and Methods

Lysozyme (Lys), bovine serum albumin (BSA), dinitrophenyl hydrazine (DNPH), glycine, *tris* (hydroxymethyl) aminomethane, Tween-20, ammonium persulphate, sodium dodecyl sulphate (SDS), para-nitrophenyl phosphate, goat anti-human IgG alkaline phosphatase conjugate (ALP), and Coomassie brilliant blue-R250 (CBB) were purchased from Sigma-Aldrich, St. Louis, Missouri, USA. Recombinant S1-RBD-protein and anti-R-C19-S1-RBD IgG (MyBioSource, San Diego, CA, USA). Enzyme-linked immunosorbent assay (ELISA) polystyrene microtiter flat-bottom (96-well) plates were sourced from Nunc (Sigma-Aldrich, St. Lois, Missouri, USA). All chemicals were of analytical grade. 

### 2.1. Human Serum Samples

Normal human sera were obtained from individuals who participated in the study as volunteers in the Hail region, KSA. Volunteer consent was obtained before the collection of samples for this study. Ethics approval for this study was given by the Research Ethics Committee, University of Hail, as protocol H-2021-122. All of the volunteers (age 21–60 years) gave samples one month after receiving the second dose of the COVID-19 vaccine. PCR tests were conducted for all volunteers to rule out positivity. It was also confirmed that the volunteers had not been infected with SARS-CoV-2 prior to the study. The psychological status of the volunteers was considered, and subjects were divided into ten groups (each group consisted of 15 volunteers) as either being depressed or not. They were also grouped as either smokers or non-smokers. Each group consisted of 15 samples from different subjects. The groups are as follows; males who were non-depressed and non-smokers (M), females who were non-depressed and non-smokers (F), males who were non-smokers but depressed (M-D), females who were non-smokers but depressed (F-D), males who were smokers but non-depressed (M-S), females who were smokers but non-depressed (F-S), males who were depressed and smokers (M-D-S), and females who were depressed and smokers (F-D-S).

We adopted a slightly modified version of a questionnaire [35] to screen the volunteers to determine their levels of depression. Depression was assessed using criteria such as mood, lack of satisfaction, feelings of failure, overall pessimism, sense of guilt, suicidal ideas, crying, feelings of irritation, social withdrawal, body image issues, work inhibition, sleep disturbances, fatigue, loss of appetite and weight loss [24]. In this study, the diagnosis of ‘depression’ was made with the consultation of a clinician in the Department of Medicine, King George’s Medical College, Lucknow, India.

Cigarette-smoking history was assessed based on a self-reported questionnaire filled out by subjects and completed before experimental work was started on the samples. Data analysis from questionnaires led to the classification of individuals who smoked ≥4 cigarettes per day as smokers in this study. Individuals who smoked ≤3 cigarettes were not included in this study. 

Fasting blood glucose (FBG) estimations and the determination of glycated hemoglobin (HbA1c) levels were assessed using well-known prescribed methods (glucose oxidase method and capillary electrophoresis method, respectively) regularly used in the clinics. Fresh blood samples were used in these assays. Basal metabolic rates (BMR) for all of the samples were calculated using online tool.

### 2.2. *Modification of BSA* and Lysozyme by ROS

An in vitro ROS modification has been carried out for BSA and Lys proteins using a previously published method [36] An aqueous solution of BSA and Lys (1 mg/mL) in PBS, pH 7.4, was irradiated under 254 nm UV light for 20–30 min at room temperature in the presence of hydrogen peroxide (10 mM). After 30 min of the reaction, all samples were dialysed against PBS, pH 7.4. Protein concentration was determined spectrophotometrically using E1%1 cm = 5.3 M^−1^ cm^−1^ at 280 nm [37], as well as by Nanodrop. 

### 2.3. Structural Modifications

Ultraviolet absorption spectroscopy: The ultraviolet spectra of native and modified protein samples (150 µg/mL) were estimated within the wavelength range of 200–420 nm on a UV–spectrophotometer. 

Tryptophan fluorescence spectroscopy: The fluorescence of tryptophan residues in native and modified protein samples was estimated at an excitation wavelength of 285 nm. The emission for all of the samples was recorded over the range of 290 to 440 nm [38]. For the analysis, the concentration of protein samples was 100 mM. All readings were measured on a Hitachi model F2700 spectrofluorometer (Japan).

### 2.4. Detection of Serum MDA Contents 

Detection of the malondialdehyde (MDA) content can reflect the level of lipid peroxidation in cells and indirectly reflect the level of cellular damage. Colorimetric assay kits were used according to the manufacturer’s instructions (Elabscience, Houston, Texas, USA). Fresh blood samples were stored in a tube with anticoagulant and centrifuged at 14,000 rpm for 10 min at 4 °C. Clear plasma without any white blood cells and platelets were separated. The plasma samples were stored at −80 °C for up to a month. All of the samples were measured at an OD of 532 nm, with 1 cm optical path cuvette, and the values were given as nmol/mL.

The amount of MDA was calculated using the given formula:

MDA = ∆A1 × c

       ∆A2

ΔA_1_ represents OD of sample—OD of control
ΔA_2_ represents OD of standard—OD of blank
C is the concentration of standard (10 nmol/mL)

### 2.5. Cytokines IFN-γ and TNF-α

Cytokines IFN-γ and TNF-α were estimated in serum samples based on quantitative sandwich immunoassay (R&D System, Minneapolis, MN, USA) with a sensitivity of less than 0.5 pg/mL for IFN-γ and 0.7 pg/mL for TNF-α. Samples were assayed in triplicate.

### 2.6. Determination of Protein Bound Carbonyl Contents

Oxidative stress is associated with COVID-19 infection [39] and depression [40]. It is a hallmark for oxidative stress and protein oxidation. Thus, it is an important factor for investigation. The protein-bound carbonyl content (CC) from the sera of subjects were analyzed according to Levine et al. [41]. All results were calculated as the number of nanomoles of carbonyl groups per milligram of sample protein, using the equation: ε379 nm = 22,000 M^–1^ cm^–1^ [42].

### 2.7. Direct and Inhibition ELISA

Direct binding and inhibition ELISA have been employed for the analysis of serum immunoglobulin G (IgG), using previously published methods [25,26,43]. Serum samples were analyzed for the detection of antibodies (IgG) against S1-RBD. A standard curve was prepared using antigen S1-RBD vs. IgG against S1-RBD. Levels of serum autoantibodies (IgG) were also analyzed against in vitro ROS modified proteins (Lys and BSA). 

### 2.8. Statistical Evaluation

Data are presented as mean±SD. Multiple comparisons between data were made using the software OriginPro 11, followed by the Student's *t*-test. The *p*-values < 0.05 were considered to indicate statistical significance. 

## 3. Results

### 3.1. Characterization of ROS-HSA by Spectroscopic Studies

**Figure 1:**
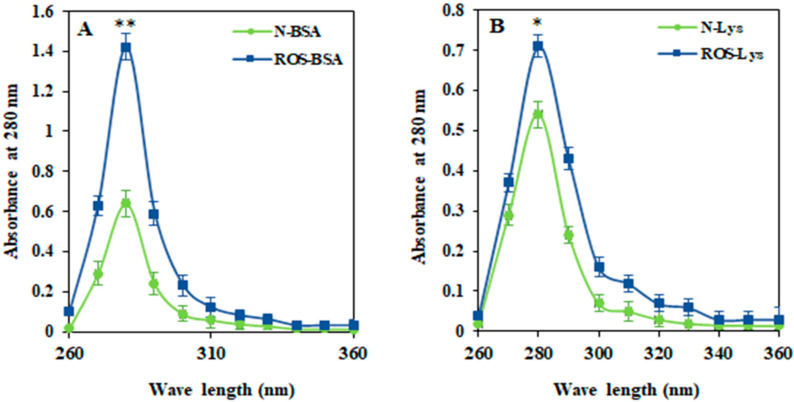
UV spectra of native and free-radical-modified proteins BSA (**A**) and Lys (**B**). The comparison of UB absorbance intensities between native BSA and ROS-BSA showed ** *p* < 0.01. Similarly, native Lys and ROS-Lys exhibited * *p* < 0.05.

ROS-induced modifications in Lys and BSA were characterized by spectroscopic analysis. A UV spectral study of ROS-modified proteins (ROS-Lys and ROS-BSA) showed significant (56.8%, *p* < 0.01 and 29%, *p* < 0.05) increase in hyperchromicity compared to non-modified (Lys and BSA) proteins at a wavelength of 280 nm (Figure 1A,B, respectively). The UV spectra of modified proteins exhibited the unfolding of biomolecules.

**Figure 2:**
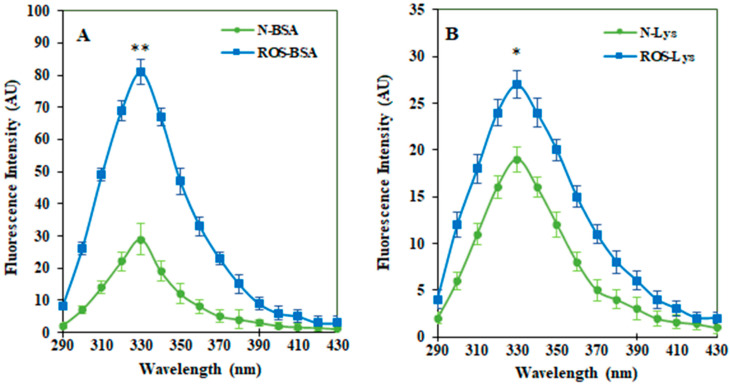
Tryptophan-specific fluorescence of native and free-radical-modified proteins BSA (**A**) and Lys (**B**). The comparison of tryptophan-specific intensities between native BSA and ROS-BSA showed ** *p* < 0.01. Similarly, native Lys and ROS-Lys exhibited * *p* < 0.05.

To study a site-specific structural change in proteins (Lys and BSA) due to free radical damage, we opted for tryptophan-specific fluorescence analysis, as Tryptophan *λ*_max_ is found to be very sensitive to changes in its local environment. Tryptophan-specific fluorescence for both native and modified proteins were recorded within the wavelength range of 290 to 430 nm. A significant increase in the tryptophan-specific fluorescence of ROS-Lys (22.79%, *p* < 0.05) and ROS-BSA (68.44%, *p* < 0.01) was observed compared to the non-modified respective proteins (Figure 2A,B, receptively). 

### 3.2. Biochemical and Immunological Analysis in Serum Samples

#### Proinflammatory Cytokines in Serum Samples

Proinflammatory cytokines IFN-γ and TNF-α were estimated in serum samples of all of the subjects from different groups (Table 1). The levels of IFN-γ (7.4 ± 0.73 pg/mL; *p* < 0.001) and TNF-α (1.37 ± 0.18 pg/mL; *p* < 0.001) increased significantly in female subjects who were smokers and depressed, as compared to female subjects who were either non-smokers and non-depressed or female subjects who were depressed or smokers. Moreover, these cytokine levels were also higher in F-D-S compared to M-D-S (IFN-γ; 6.6 ± 0.65 pg/mL and TNF-α; 1.29 ± 0.19 pg/mL). Interestingly, higher levels of IFN-γ and TNF-α were found in F-D subjects compared to M-D.

### 3.3. Serum Carbonyl Content

The generation of protein-bound carbonyl content is a well-known oxidative stress marker for protein oxidation. The dinitrophenylhydrazine (DNPH) reaction was used to estimate protein carbonyl content. The estimation of carbonyl content was conducted in all samples from each group (Table 1). Significantly higher amounts of carbonyl content (nmol/mg protein) were detected in subjects from the F-D-S group (2.42 ± 0.38) followed by M-D-S (2.11 ± 0.34), F-D (1.13 ± 0.17), M-D (0.88 ± 0.13), M-S (0.99 ± 0.16) and F-S (0.89 ± 0.11). A lower amount of carbonyl content was detected in male (0.71 ± 0.08) and female (0.68 ± 0.07) subjects who reported no signs of psychological stress and were also non-smokers. Subjects from the F-D-S group showed the highest amount of carbonyl compounds compared to F-D and F-S subjects. Similar trends in serum carbonyl content were observed in groups that included males. Moreover, it was observed that F-D-S subject samples contained significantly higher (*p* < 0.01) amounts of carbonyl groups compared to M-D-S subjects.

### 3.4. Serum MDA Levels

Subjects from all of the groups were analyzed for serum MDA levels (Table 1). The mean serum MDA level in group F-D-S was 2.19 ± 0.42 nmol/mL, which was significantly higher than F-D (1.47 ± 0.27 nmol/mL) and F-S (0.84 ± 0.19 nmol/mL). Similarly, the mean serum MDA levels of the M-D-S group was 1.81 ± 0.31 nmol/mL which was higher than the mean value for subjects from the M-D (1.21 ± 0.21 nmol/mL) and M-S (0.87 ± 0.16 nmol/mL) groups. Compared to male subjects with similar conditions, female subjects classified as either depressed, or both depressed and smokers, exhibited higher MDA levels in serum.

### 3.5. Clinical Characterization Analyses

FBG and HbA1c have been identified as predictors of COVID-19 severity and associated mortality [44,45]. Both have also been implicated in patients with depressive symptoms [46,47]. Thus, we selected these clinical investigations in our study. Subjects from the F-D-S group showed significant increase only in mean FBG levels compared to other groups. However, no significant change was observed in glycated hemoglobin in all the cohorts (Table 1). A significantly high BMR was found in smokers compared to the non-smokers among the same gender groups. Female subjects exhibited a lower BMR compared to the males.

### 3.6. Direct Binding ELISA

**Figure 3:**
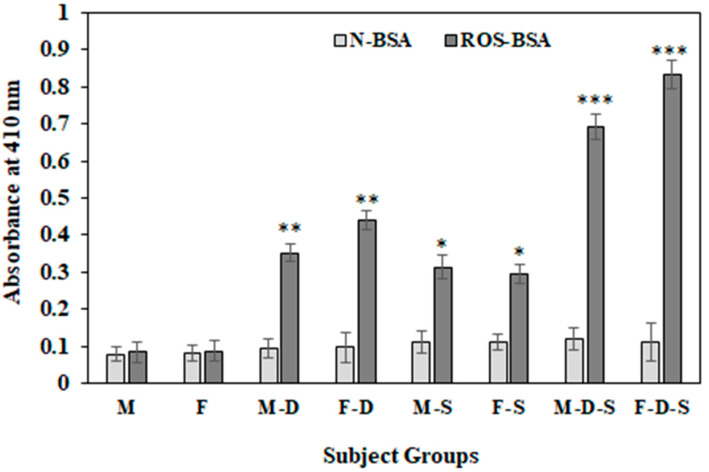
Detection of serum autoantibodies against native and ROS-BSA using direct binding ELISA. ELISA plates were coated with 5 µg/mL antigens. Each group consisted of 15 subjects. Values are given as mean±SD. The t test was adopted for comparison between the two groups, and significance is defined as * *p* < 0.05, ** *p* < 0.01, *** *p* < 0.001. The comparison of autoantibodies against ROS-BSA between group M with groups M-D, M-S and M-D-S showed *p* values < 0.01, <0.05 and <0.001, respectively. The comparison of autoantibodies against ROS-BSA between group F and groups F-D, F-S and F-D-S showed *p* values < 0.01, <0.05 and <0.001, respectively. No significance was observed among the groups for native BSA.

Direct binding ELISA was used to identify the levels of the serum IgGs against various antigens investigated in this study. The oxidation of protein sera from different subject groups was investigated by categorizing subjects as normal individuals or psychologically depressed individuals who were either smokers or non-smokers. Sera samples from all different groups were tested against both N-BSA and ROS-BSA using direct ELISA (Figure 3). The highest levels of serum autoantibodies against ROS-BSA were observed in the F-D-S group (OD; 0.833 ± 0.051) compared to the remaining groups. Levels of serum autoantibodies against ROS-BSA in subjects from the F-D group (OD; 0.439 ± 0.039) were higher than in subjects from the M-D group (OD; 0.352 ± 0.027). 

**Figure 4:**
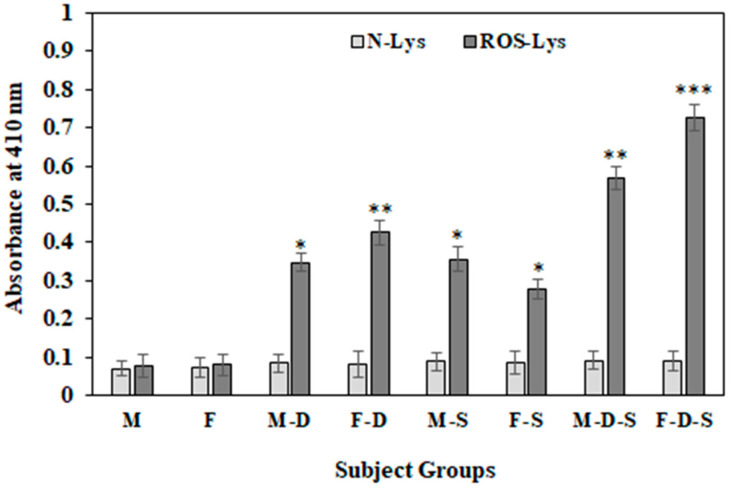
Detection of serum autoantibodies against native and ROS-Lys using direct binding ELISA. ELISA plates were coated with 5 µg/mL antigens. Each group consisted of 15 subjects. Values are given as mean±SD. The t test was adopted for the comparison between the two groups, and significance is defined as * *p* < 0.05, ** *p* < 0.01, *** *p* < 0.001. The comparison of autoantibodies against ROS-Lys between group M with groups M-D, M-S and M-D-S showed *p* values < 0.05, <0.05 and <0.01, respectively. The comparison of autoantibodies against ROS-Lys between group F and groups F-D, F-S and F-D-S showed *p* values < 0.01, <0.05 and <0.001, respectively. No significance was observed amongst the groups for native Lys.

Circulatory autoantibodies were estimated in samples from all groups against another free radical modified and non-modified protein i.e., Lys (Figure 4). A similar pattern of the serum autoantibodies levels against ROS-Lys and N-Lys were observed, as shown by ROS-BSA and N-BSA. Subjects from the F-D-S group showed the highest recognition (OD 0.725 ± 0.034) of circulatory antibodies against ROS-Lys, followed by M-D-S (OD 0.568 ± 0.032), F-D (OD 0.425 ± 0.033), M-S (OD 0.356 ± 0.031), M-D (OD 0.347 ± 0.024) and F-S (OD 0.276 ± 0.026) (Figure 4). Subjects from groups M and F showed very low recognition with ROS-BSA. Conversely, with N-BSA, no appreciable bindings were observed in any of the groups.

**Figure 5:**
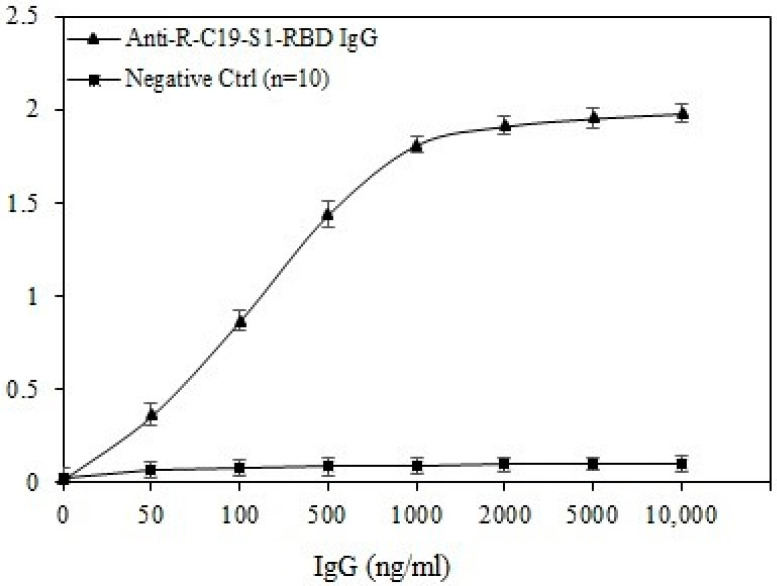
Standard graph between COVID-19 virus protein antigen S1-RBD vs. IgG against S1-RBD. For the positive control, anti-R-C19-S1-RBD IgG was used. Ten serum samples (IgG) from pre-pandemic subjects served as negative control. ELISA plates were coated with an antigen (S1-RBD) concentration of 5 μg/mL. Each sample was run in triplicate under similar conditions.

A standard curve between COVID-19 virus protein antigen S1-RBD vs IgG against S1-RBD was prepared to estimate a cut off measurement (Figure 5). In this assay anti-R-C19-S1-RBD IgG was used as positive control. Negative controls (n = 10) used in this curve are human serum samples before the COVID-19 pandemic, based on these negative samples values (average) in the standard curve, a cut of measurement was found to be 0.11 OD. OD values above that were considered positive. 

**Figure 6:**
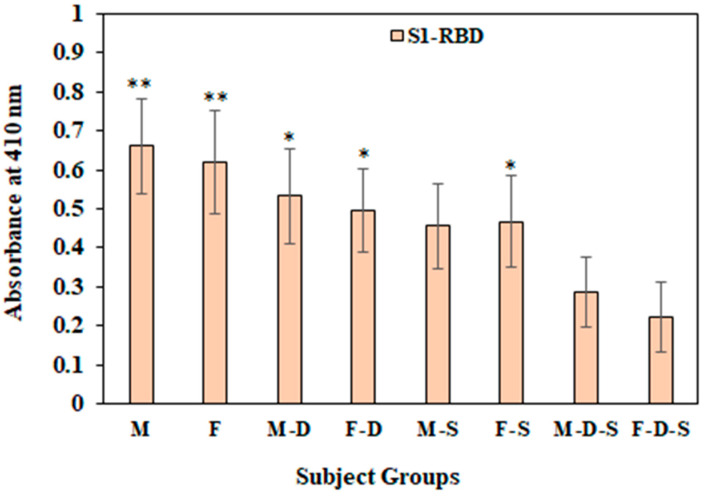
Detection of serum autoantibodies against ROS modified proteins (BSA and Lys) and S1-RBD antigen using direct binding ELISA. ELISA plates were coated with 5 µg/mL antigens. Each group consisted of 15 subjects. Values are given as mean ± SD. The t test was adopted for a comparison between the two groups, and significance is defined as * *p* < 0.05, ** *p* < 0.01. The comparison of S1-RBD-Abs between group M-D-S with groups M, M-D and M-S showed *p* values < 0.01, <0.05 and *p* > 0.05, respectively. The comparison of S1-RBD-Abs between group F-D-S and groups F, F-D, and F-S showed *p* values < 0.01, <0.05 and <0.05, respectively.

Level of antibodies were also detected in all of the groups using the S1-RBD antigen in direct binding ELISA assay (Figure 6). Significantly (*p* < 0.01) high levels of S1-RBD-Abs were observed in group M (0.66 ± 0.121) and F (0.618 ± 0.133) compared to M-D-S (0.287 ± 0.09) and F-D-S (0.221 ± 0.089) groups. Furthermore, F-D (0.497 ± 0.107) and F-S (0.467 ± 0.118) groups exhibited significantly (*p* < 0.05) higher levels of S1-RBD-Abs compared with group F-D-S (0.221 ± 0.089). 

However, in male subjects only, the M-D (0.533 ± 0.122) group and not M-S (0.456 ± 0.109) showed significantly higher (*p* < 0.05) levels of S1-RBD-Abs compared with M-D-S group subjects (0.287 ± 0.09) (Figure 6). Importantly, serum S1-RBD-Abs were found to be low in F-D-S group subjects as compared to M-D-S; however, the difference was non-significant. Furthermore, there was a significant decrease in the levels of these S1-RBD-Abs in subjects who were under psychological depression and were smokers. Our results indicate that the combined effects of depression and smoking habit are more pronounced in female subjects as compared to male subjects. Inflammatory cytokines such as IFN-γ and TNF-α may trigger depression in humans. In various diseases such as cancer, hormonal diseases and infectious diseases, as well as autoimmune diseases, levels of proinflammatory cytokines are linked directly to the risk of stress or depression.

**Figure 7:**
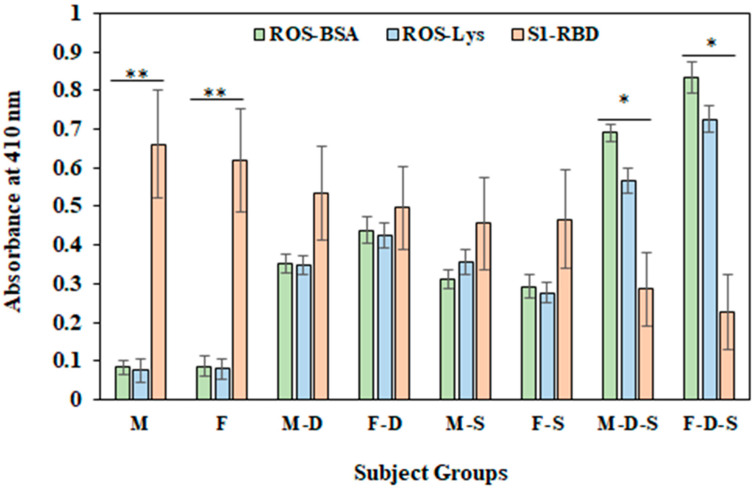
Detection of serum autoantibodies against ROS modified proteins (BSA and Lys) and S1-RBD antigen using direct binding ELISA. ELISA plates were coated with 5 µg/mL antigens. Each group consisted of 15 subjects. Values are given as mean ± SD. The t test was adopted for the comparison between the two groups, and significance is defined as * *p* < 0.05, ** *p* < 0.01.

A common figure (Figure 7) represents the levels of serum autoantibodies against free-radical-modified protein antigens (ROS-BSA and ROS-Lys) compared with the levels of serum S1-RBD-Abs. 

In groups M (0.661 ± 0.121) and F (0.618 ± 0.133), significantly high levels of the S1-RBD-Abs antigen were observed compared to levels of autoantibodies against ROS-modified BSA (0.083 ± 0.031 and 0.086 ± 0.028, for group M and F group respectively) and Lys (0.076 ± 0.021 and 0.079 ± 0.026, for group M and F group, respectively). Conversely, in group M-D-S and F-D-S, significantly higher (*p* < 0.05) levels of autoantibodies against ROS-modified BSA and Lys were observed compared to the levels of S1-RBD-Abs (Figure 7). This result demonstrates that during the pandemic period, in subjects without any sign of psychological complications, the levels of S1-RBD-Abs are present in respectable amounts in both male and female subjects. However, in subjects with depression and smoking habits, there is a decrease in the levels of S1-RBD-Abs, with a significant increase in autoantibodies against ROS-modified protein antigens (ROS-BSA and ROS-Lys).

### 3.7. Inhibition ELISA

The binding specificity of serum autoantibodies and S1-RBD-Abs were analyzed using competitive ELISA assays. Free-radical-modified proteins (BSA and Lys), as well as the SARS-CoV-2 virus antigen S1-RBD, were used as inhibitors in these immune assays (Table 2). Values for each group are given as mean maximum percent inhibition (MMPI) with standard deviation. These values only include MMPI values from subjects who exhibited greater than 10% inhibition at maximum concentrations (10 μg/mL) of inhibitors. For the non-depressed and non-smoker subjects from both males and females, inhibition of less than 8% was detected against both antigens. 

For ROS-modified antigens ROS-BSA and ROS-Lys, the highest MMPI observed in the F-D-S group were 77.7 ± 6.2 MMPI and 73.8 ± 6.0 MMPI, respectively. In contrast, the serum IgG from subjects of the F-D-S group exhibited the lowest MMPI (59.8 ± 6.0) with the S1-RBD antigen. Even the number of subjects who exhibited an MMPI of greater than 10% was lowest in the F-D-S group. These results were followed by the group M-D-S. A comparison of the M-D and F-D groups showed that depression alone exerts an effect on the serum IgG levels against all of the antigens used in this study. Increased serum IgG levels against ROS-modified proteins were observed; however, there was a decrease in Anti-S1-RBD Abs. The effect of smoking showed minimum differences in serum IgG levels among the groups (M-S and F-S). These results showed oxidative and psychological stress conditions induced serum autoantibodies against free-radical-modified protein antigens, while serum S1-RBD-Abs were decreased. 

## 4. Discussion

SARS-CoV-2 attacks the lower respiratory tract, activating immune cells and causing the release of increased amounts of cytokines and chemokines, known as a ‘cytokine storm’ [5]. These events are often accompanied by severe pneumonia, affecting several organs. Varying signs and symptoms together with psychological harm were observed in COVID-19 patients [48,49]. Other than the polymerase chain reaction test for COVID-19 infection, alterations in liver enzymes, lymphocyte levels, and C-reactive proteins were apparent. On 1st April 2020, U.S. Food and Drug Emergency Use Administration granted the use of a SARS-CoV-2 antibody test [50]. This test shows the humoral immune response against SARS-Cov-2. Different platforms such as adenovirus-based, RNA-based, DNA-based, lipid nanoparticles, etc., were used to develop several SARS-CoV2 vaccines. However, their long-term efficacy and safety are still to be confirmed [51].

Serum S1-RBD-Abs are the first line of defense in the fight against SARS-CoV-2 infection and predictive of immune protection. The extent of resistance offered against various COVID-19 variants is dependent on the durability and levels of these S1-RBD-Abs in circulation. A study suggests that many host factors contribute to the changing neutralization titers such as age, gender and comorbidities [52]. It has been observed that anxiety and psychological stress increased manifold in individuals during the COVID-19 pandemic [53,54]. The antioxidant function of *N. sativa* has been studied recently, and in an in silico study, it was proven that the bioactive molecules of *N. sativa* can inhibit the binding of the SARS-CoV-2 ligand and its receptor [55].

We hypothesized that an increase in psychological and oxidative stress might favor the production of circulatory autoantibodies. A concurrent waning in the levels of serum S1-RBD-Abs was also predicted. We tested our hypothesis, using the well-established immunoassay ‘ELISA’. Direct binding and inhibition ELISA assays were used to analyze the levels of the antibodies and their specificity towards respective antigens. Both the given immunoassays were also used to identify the levels of serum IgG against the S1-RBD antigen and their specificity towards the antigen. Two different in vitro modified free radical proteins predominantly found in circulation (albumin and lysozyme) were used as antigens, and autoantibodies were screened against ROS-BSA and ROS-Lys proteins. The levels of these autoantibodies were then compared with the antibodies against COVID-19 viral antigen S1-RBD. 

Spectroscopic studies for UV–vis and tryptophan-specific fluorescence revealed significant modification in both proteins (BSA and Lys). These changes can be attributed to the exposure of chromophobic groups in the protein structure [36,37]. Any change in tryptophan-specific fluorescence provides critical insight into site-specific structural changes due to oxidative stress. These effects of free radicals on protein molecules suggest potential in vivo protein structural alterations. These modified protein molecules were used as antigens to screen serum autoantibodies in subjects from eight different groups as defined in methods.

The demographic, biochemical, immunological and psychological comparisons were made among the groups based on FBG, HbA1_C_, serum MDA and carbonyl content, proinflammatory cytokines (IFN-γ and TNF-α), smoking habits and psychological stress, together with the levels of serum autoantibodies and S1-RBD-Abs. It has been observed that people with mental disorders smoke 2–4 times more than healthy individuals. Stress and anxiety increase smoking frequency. Therefore, it is possible that the pandemic has affected smoking behavior [56]

Out of two blood sugar-level parameters FBG and HbA1c, only FBG has been found to be implicated in female patients with depression, which corresponds to previous findings [57]. The BMR increases in smokers compared to non-smokers [58]. A significant increased levels in BMR were detected in smokers compared to non-smoker subjects in gender-based groups. In females, BMR is lower than in age-matched male subjects [59].

Levels of pro-inflammatory cytokines are linked directly to the risk of stress or depression in various diseases such as cancer, hormonal diseases, infectious diseases and autoimmune diseases [60,61,62,63,64,65,66]. The correlation of IFN-γ and TNF-α with the level of depression is dose-dependent [67,68].

Carbonyl contents were found to be significantly higher in subjects who were depressed. Furthermore, difference in gender is a contributory factor, with more females experiencing depression compared to males [69]. Results consistently showed higher levels of carbonyl content in female subjects’ serum samples compared to male counterparts. Mood disorders are influenced by neuroendocrine abnormalities and neurotransmitter dysregulation, as well as disturbances in circadian rhythm. Fluctuating levels of sex hormones, estrogen and progesterone predispose women to mood disorders [70]. 

Depression is linked to a decrease in levels of serum antioxidants, such as vitamin E, albumin, zinc, tryptophan, tyrosine, glutathione and CoQ10 [40,71,72], and an increase in levels of a well-known oxidative stress marker, MDA. Levels of MDA were significantly higher in individuals who were suffering from major depression than in healthy individuals (*p* < 0.0001). A concomitant significant decrease (*p* < 0.0001) in the levels of major antioxidants (ascorbic acid and SOD) was observed in such patients compared to healthy individuals [73]. Thus, the failure of the removal of excessive free radicals due to non- or inadequate activity of antioxidant defense mechanisms may lead to oxidative stress injury. There is a fine balance between the oxygen demand of the brain and higher lipid content. The slightest imbalance between these two conditions might induce oxidative stress, which is a leading contributory factor in the pathogenesis of inflammatory and neuro-psychiatric disorders [73,74]. 

Pro-oxidant factors increase in smokers due to a decrease in the levels of antioxidants. Consequently, oxidative stress is induced in vascular elements including red blood cells and platelets [75]. An upregulation of HIF-1α gene expression in smokers causes the transcription of endothelial nitric oxide synthase and erythropoietin genes under oxidative/nitrosative stress conditions [75]. These findings were consistent with our results; compared to male subjects with the same conditions, female subjects suffering from depression and with a history of smoking had significantly higher levels of MDA and carbonyl content (*p* < 0.01). Also, when comparing among only female groups, individuals with depression and smoking history exhibited higher carbonyl content than subjects who were only depressed or only smokers or subjects who had neither of these conditions. 

Strong neutralizing antibody responses against the spike glycoprotein of the SARS-CoV infections in 2002–2003 protected infected hosts from severe disease [76]. Similarly, for the current pandemic, it has been hypothesized that the production of antibodies to SARS-CoV-2 would be critical in limiting disease progression. Recent studies have indicated that S1-RBD-Abs found in convalescent COVID-19 patient plasma supported fast recovery when administered to critically ill patients [77,78,79]. Some of the studies proved the presence of autoantibodies against ANAs were detected in COVID-19 patients, especially in severe and critically ill patients. Moreover, in these patients, the ANA positivity rate ranges from 33.3 to 50% [29,30,31,32]. Inflammatory conditions are associated with autoantibody production in depressed subjects with various physiological conditions [25,26,34]. Patients with systemic lupus erythematosus, rheumatoid arthritis and type 1 diabetes showed increased levels of autoantibodies [25,26,33,34]. 

An increase in free radical generation and the consequent imbalance of redox homeostasis are involved in many respiratory viral infections. Viral infections can induce the secretion of pro-inflammatory cytokines, the activation of innate immunity, enhanced lipid peroxidation, the production of free radicals and the concomitant depletion of glutathione (GSH) and inhibiting nuclear factor (erythroid-derived 2)-like 2 (NRF2) in epithelial cells of the respiratory tract [77]. Previous findings suggest that innate immunity and oxidative stress are involved in severe lung necrosis in SARS-CoV infection [80]. A recent study on patients of COVID-19 who were admitted in intensive care were found to exhibit decreased levels of circulatory antioxidants (like β-carotene, γ-tocopherol, vitamin C and GSH) and showed enhanced lipid peroxidation and oxidative stress [81].

In a recent study, increased levels of autoantibodies were detected against an oxidative stress and lipid oxidation marker, MDA, in COVID-19 patients compared to healthy individuals [82]. Excessive oxidative stress may be a potential reason for the production of autoantibodies. Oxidative stress might cause cellular damage, which leads to the release of intracellular antigens, together with the formation of circulatory neo-antigens from self-proteins, modified by excessive free radicals. Our findings indicate an excessive titer of autoantibodies and a decrease in the titer of S1-RBD-Abs in subjects who were depressed and smokers. Female subjects with both these conditions showed more autoantibodies compared to males.

Limitations of this study: (1) the number of recruited participants in this study was small; (2) limited clinical investigations for the participants were available; and (3) socio-economic details of the participants were not available.

## 5. Conclusions

Subjects with depression and smoking habits are exposed to excessive oxidative stress, which leads to unregulated redox homeostasis, causing damage to circulatory proteins. These damaged blood proteins induce the formation of neo-antigens, which lead to the generation of autoantibodies. Together with other clinical changes, increased oxidative stress and proinflammatory cytokines (IFN-γ and TNF-α) can plausibly be implicated in immune balance shifts towards a decrease in COVID-19-specific antibodies. Future studies including more healthy subjects and SARS-CoV-2-infected patients should include screening for diverse antigens to identify specific circulatory autoantibodies. Detailed clinical investigations and socio-economic details of participants could lead to insights, facilitating the better management of COVID-19, especially for patients under major psychological stress.

## Figures and Tables

**Table 1:** Clinical characterizations of individuals based on gender and smoking habits.

Groups (21–60 Years) *n* = 15	Age (Years ± SD)	BMR (cal/sq.m/hr)	FBG (mg/dL)	HbA1_C_ (%)	IFN-γ (pg/mL)	TNF-α (pg/mL)	Smoking Duration (Years ± SD)	MDA Content nmol/mL	Carbonyl Content (nmol/mg Protein)
M	38 ± 9.3	36.3 ± 3.4	87.9 ± 8.1	5.4 ± 0.3	4.6 ± 0.41	0.95 ± 0.15	―	0.77 ± 0.17	0.71 ± 0.08
F	40 ± 10.8	32.1 ± 3.1	88.6 ± 9.5	5.5 ± 0.3	4.5 ± 0.43	0.96 ± 0.14	―	0.79 ± 0.16	0.68 ± 0.07
M-D	39 ± 8.5	37.5 ± 3.9	90.5 ± 7.5	5.6 ± 0.3	5.3 ± 0.48 *	1.06 ± 0.11 *	―	1.21 ± 0.21 *	0.88 ± 0.13 *
F-D	37 ± 11.1	32.8 ± 4.1	91.1 ± 6.6	5.5 ± 0.4	5.6 ± 0.49 *	1.16 ± 0.18 *	―	1.47 ± 0.27 *	1.13 ± 0.17 **
M-S	41 ± 10.2	41.4 ± 3.8 *	88.7 ± 7.8	5.4 ± 0.4	4.8 ± 0.51	1.0 ± 0.16	19.2 ± 9.2 *	0.87 ± 0.16	0.99 ± 0.16 *
F-S	38 ± 12.4	35.8 ± 3.7 *	89.4 ± 8.3	5.4 ± 0.3	4.7 ± 0.52	1.03 ± 0.12	8.5 ± 4.1	0.84 ± 0.19	0.89 ± 0.11 *
M-D-S	41 ± 11.7	42.8 ± 4.4 *	93.8 ± 7.7	5.6 ± 0.3	6.6 ± 0.65 ***	1.29 ± 0.19 **	18.9 ± 5.4 *	1.81 ± 0.31 **	2.11 ± 0.34 ***
F-D-S	40 ± 9.9	36.9 ± 3.9 *	94.1 ± 6.9 *	5.7 ± 0.3	7.4 ± 0.73 ***	1.37 ± 0.18 **	12.9 ± 6.3	2.19 ± 0.42 ***	2.42 ± 0.38 ***

Each group consist of 15 subjects. All tests for each serum sample were run in triplicate. All vales are given as mean ± standard deviation (SD). * *p <* 0.05, ** *p*  <  0.01, *** *p* < 0.001.

**Table 2:** Inhibition ELISA assay for all of the subjects from different groups against the S1-RBD antigen and various native and ROS-modified protein antigens.

Groups	Anti-ROS-BSA-Ab	Anti-ROS-Lys-Ab	Anti-S1-RBD-Ab
M	6.1 ± 1.4	7.7 ± 0.8	10 * (86.9 ± 1.7)
F	6.6 ± 1.6	7.9 ± 1.0	10 * (87.9 ± 1.9)
M-D	10 * (38.7 ± 3.3)	9 * (35.8 ± 3.9)	11 * (80.8 ± 3.9)
F-D	10 * (51.5 ± 4.2)	10 * (46.1 ± 4.5)	10 * (73.1 ± 4.5)
M-S	11 * (40.3 ± 4.1)	10 * (37.5 ± 3.8)	11 * (81.5 ± 3.8)
F-S	9 * (35.3 ± 3.3)	8 * (33.1 ± 3.0)	12 * (83.1 ± 3.0)
M-D-S	11 * (59.1 ± 5.5)	10 * (57.3 ± 5.3)	10 * (68.3 ± 5.3)
F-D-S	12 * (77.7 ± 6.2)	12 * (73.8 ± 6.0)	9 * (59.8 ± 6.0)

* Represents the number of subjects who showed >10% inhibition against either of the antigens (ROS-BSA, ROS-Lys and S1-RBD). Each group consisted of 15 samples. Range of antigens concentrations used were 0 to 10 μg/mL.

## Data Availability

The data that support the findings of this study are available on request from the corresponding author. The data are not publicly available due to privacy or ethical restrictions.
